# Exploring Bacterial Organelle Interactomes: A Model of the Protein-Protein Interaction Network in the Pdu Microcompartment

**DOI:** 10.1371/journal.pcbi.1004067

**Published:** 2015-02-03

**Authors:** Julien Jorda, Yu Liu, Thomas A. Bobik, Todd O. Yeates

**Affiliations:** 1 UCLA-DOE Institute for Genomics and Proteomics, Los Angeles, California, United States of America; 2 Roy J. Carver Department of Biochemistry, Biophysics and Molecular Biology, Iowa State University, Ames, Iowa, United States of America; 3 Molecular Biology Institute, University of California, Los Angeles, Los Angeles, California, United States of America; 4 Department of Chemistry and Biochemistry, University of California, Los Angeles, Los Angeles, California, United States of America; Boston University, United States of America; Boston University, UNITED STATES

## Abstract

Bacterial microcompartments (MCPs) are protein-bound organelles that carry out diverse metabolic pathways in a wide range of bacteria. These supramolecular assemblies consist of a thin outer protein shell, reminiscent of a viral capsid, which encapsulates sequentially acting enzymes. The most complex MCP elucidated so far is the propanediol utilizing (Pdu) microcompartment. It contains the reactions for degrading 1,2-propanediol. While several experimental studies on the Pdu system have provided hints about its organization, a clear picture of how all the individual components interact has not emerged yet. Here we use co-evolution-based methods, involving pairwise comparisons of protein phylogenetic trees, to predict the protein-protein interaction (PPI) network governing the assembly of the Pdu MCP. We propose a model of the Pdu interactome, from which selected PPIs are further inspected via computational docking simulations. We find that shell protein PduA is able to serve as a “universal hub” for targeting an array of enzymes presenting special N-terminal extensions, namely PduC, D, E, L and P. The varied N-terminal peptides are predicted to bind in the same cleft on the presumptive luminal face of the PduA hexamer. We also propose that PduV, a protein of unknown function with remote homology to the Ras-like GTPase superfamily, is likely to localize outside the MCP, interacting with the protruding β-barrel of the hexameric PduU shell protein. Preliminary experiments involving a bacterial two-hybrid assay are presented that corroborate the existence of a PduU-PduV interaction. This first systematic computational study aimed at characterizing the interactome of a bacterial microcompartment provides fresh insight into the organization of the Pdu MCP.

## Author Summary

Many bacteria produce giant proteinaceous structures within their cells, which they use to carry out special metabolic reactions in their interior. Much has been learned recently about the individual components—shell proteins and encapsulated enzymes—that assemble together, thousands of subunits in all, to make these bacterial microcompartments or MCPs. However, in order to carry out their biological functions, these systems must be highly organized through specific protein-protein interactions, and such a higher level understanding of organization in MCP systems is lacking. In this study, we use genomic data and phylogenetic analysis to predict the network of interactions between the approximately 20 different kinds of proteins and enzymes present in the Pdu MCP. Then, we use computational docking to examine a subset of those that are predicted to involve enzymes bound to the interior surface of the shell proteins, and show that the results are consistent with recent experimental data. We further provide new experimental evidence for one of the predicted protein-protein interactions. This study expands our understanding of a complex system of proteins serving as a metabolic organelle in bacterial cells, and provides a foundation for further experimental investigations.

## Introduction

Cellular organization has long been considered to be much simpler in bacteria than in eukaryotic cells. However, accumulating evidence indicates a higher-order organization in terms of cellular compartmentalization [1–3] and cell structure [4,5]. In particular, electron microscopy and higher resolution structural studies have demonstrated that some bacteria can form polyhedral capsid-like bodies that are 80 to 150 nm in diameter [6,7]; reviewed in [8–11]. These polyhedral inclusions, known as bacterial microcompartments, are widely distributed across nearly 20% of known bacterial strains [9,12,13]. We refer here to bacterial microcompartments as MCPs; they are sometimes referred to as BMC’s, but we reserve the latter name to refer to the family of shell proteins that comprise MCP shells. As opposed to membrane bound organelles characteristic of eukaryotic cells, MCPs are exclusively proteinaceous assemblies; they consist of a thin outer protein shell enclosing a metabolically active core of enzymes, earning them the status of bacterial organelles. MCPs fulfill diverse roles: enhancement of metabolic flux in their hosted enzymatic pathway [14], confinement of toxic or volatile intermediates [15–17] and shielding of interior enzymes from reactions with reactive or competing molecules [18].

**Figure 1:**
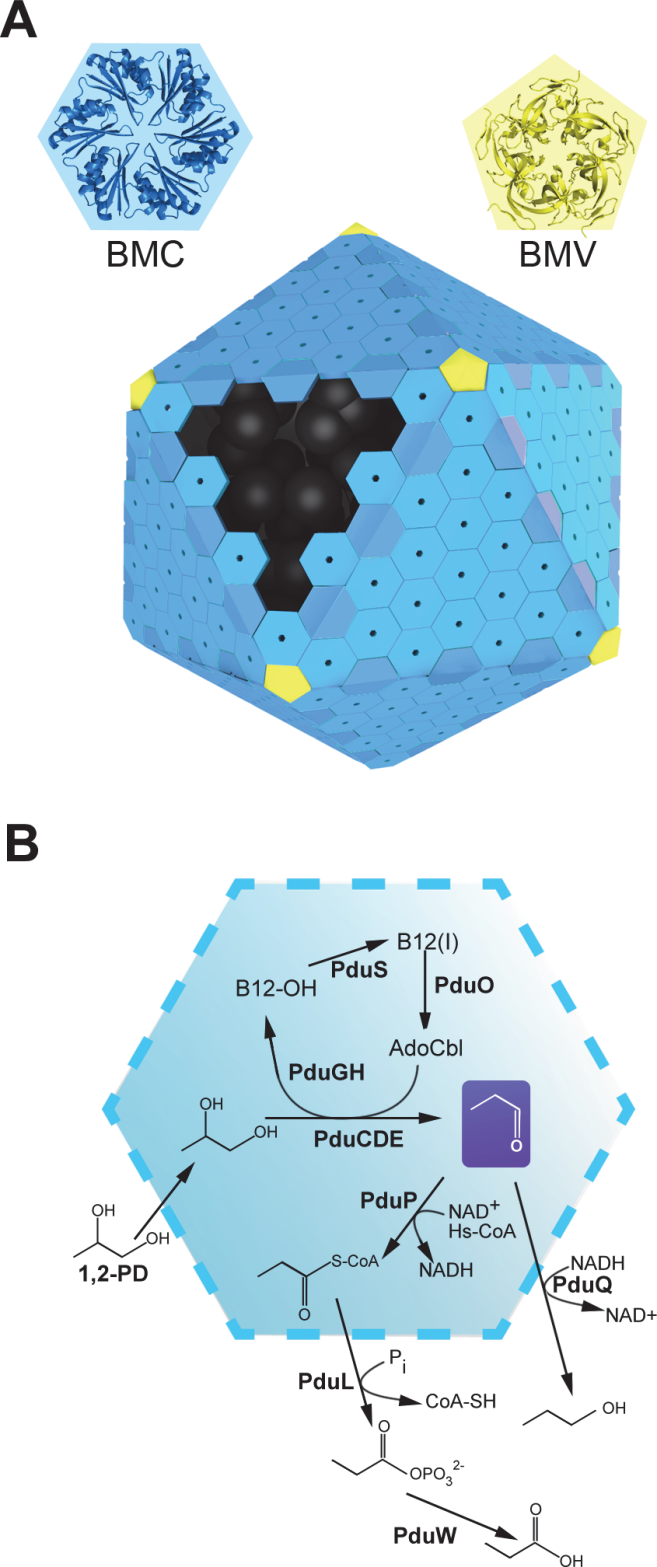
An idealized model of the Pdu MCP shell and its encapsulated pathway. The MCP shell is assembled from a few thousand copies of proteins belonging to the BMC (bacterial microcompartment) protein family. Several distinct paralogs from the BMC family are present within a single shell. BMC proteins self-assemble into cyclical hexamers (in blue). Also present in fewer copies are proteins from a distinct family, referred to as BMVs, which are pentameric proteins (in yellow) forming the vertices of the polyhedral structure. The polyhedron is shown here idealized as an icosahedron, while the Pdu MCP is typically less regular in shape. Sequentially acting enzymes (in black) carrying out the Pdu pathway are enclosed by the shell (A). The Pdu pathway degrades 1,2-propanediol to propionaldehyde via a B_12_-dependent catalytic mechanism, the aldehyde being subsequently converted to 1-propanol or propionyl-phosphate (B).

The founding member of the MCP family, the carboxysome, was first isolated 40 years ago [19]. Carboxysomes are present in some chemotrophic bacteria and probably all cyanobacteria [18,20,21]. The carboxysome serves as an organelle for carbon fixation through the encapsulation of two enzymes: carbonic anhydrase and ribulose-1,5-bisphosphate carboxylase/oxygenase (RubisCO). Several other kinds of MCPs are found dispersed across the bacterial kingdom, where they carry out metabolic pathways different from carbon fixation. These include the Pdu and the Eut microcompartment from *Salmonella* [22–24] and *E.coli* [25,26], which carry out the degradation of 1,2-propanediol and ethanolamine, respectively. These pathways rely on a similar mechanism: an initial substrate is first converted by a B_12_-dependent enzyme to give an aldehyde intermediate, which is sequestered long enough to be converted to less toxic metabolites, e.g. an alcohol and/or carboxylic acid. However, these three relatively well-characterized MCPs (carboxysome, Pdu and Eut) constitute only a subset of the entire MCP family. Recent computational and experimental studies delineate at least seven kinds of MCPs, all with different metabolic purposes [13,27–30]. The accepted three-dimensional model of the Pdu MCP and its encapsulated metabolic pathway is summarized in Fig. 1B.

Though MCPs differ substantially according to their metabolic nature, they share a number of genomic and structural characteristics. In particular, most MCP proteins are encoded within operons, which consist of multiple paralagous genes coding for the shell proteins alongside the genes for the associated enzymes. Consistent with this shared genomic signature, diverse MCPs share a similar organization and structure. Typically, each shell protein sequence is comprised by a bacterial microcompartment (BMC) domain, or sometimes two such domains duplicated in tandem. The first high resolution structures of BMC proteins shed light on the structural organization of the MCP shell [9–11,28,31–35]. A few thousand copies of these BMC proteins self-assemble into cyclic hexameric units packed side-by-side in a layer forming the essentially flat facets of the roughly icosahedral structure (Fig. 1A). The top and bottom sides of a BMC hexamer typically show distinctly different features: one face bears a central depression giving rise to a concave shape, whereas the other side is typically flatter and more polar in chemical character. Which of the two sides (convex or flat) faces inward to the MCP lumen is a question of key significance for MCP function [36–38]. Most often, the center of the hexamer is perforated by a narrow (4–7 Å) hydrophilic pore that is thought to act as a canal for molecular transport [32,38–40]. In addition to the main BMC shell proteins, other minor proteins have been found to be essential to the formation or closure of the shell. These proteins, which our group recently coined the bacterial micrompartment vertex (BMVs) proteins, assemble into pentamers suspected to close the vertices of the MCP [37,41,42] (Fig. 1A). Furthermore, a number of intriguing variations such as domain fusion, tandem duplication, circular permutation, or FeS cluster binding sites, have been revealed among the crystal structures of the paralogous BMC shell proteins [43–46]. Speculations on the roles of such variations support the idea that each type of BMC paralog has a defined role beyond simply assembling to form a physical barrier.

Interactions between the shell proteins and the encapsulated enzymes are vital for MCP function. Recent studies on the assembly of the α-type carboxysome suggest assembly of this type of MCP is initiated from the interior; the formation of enzymatic seeds precedes acquisition of the shell [47,48]. However, the processes governing the interactions between the encapsulated enzymes and the shell proteins are complex and apparently divergent between different types of MCPs. Specific interactions have been demonstrated in a few cases using pull-down assays and other experiments [36,49]. Fan *et al*. [50] first showed that short sequence extensions present at the N-terminus of numerous enzymes exist to bind enzymes to the MCP shell. A subsequent study showed that the C-terminal region of an α-carboxysomal protein (CcmN) interacted with the shell in that system [49]. Though enzyme targeting mechanisms are presumed to be widespread across the MCP systems, only a few enzyme-shell protein interactions have been specifically identified. Characterizing these interactions would open new perspectives on MCP biology and applications in synthetic biology [51,52]. Some progress has already been made along these lines. Fluorescent proteins and other proteins have been successfully directed to MCPs by appending terminal targeting peptides [29,50,53–55].

Despite knowing the identities of a few interactions between enzymes and shell proteins, atomic level detail is lacking. Attempts to isolate and determine the structures of cognate complexes have been unsuccessful. This has prompted us to undertake a computational study to develop interaction models for an MCP system. The ever-increasing genomic and structural data available for MCPs provides an unprecedented opportunity to apply computational methods to characterize the molecular networks ruling these extraordinary supramolecular machines. Over the last two decades, a handful of methods exploring genomic data have been developed for predicting functional linkages between different proteins in a cell. Popular methods such as protein phylogenetic profiles [56,57], gene fusion [58,59], gene neighborhood [60,61] or a combination of these [62–64], have been used extensively to make functional inferences about proteins. Indeed, one of our recent studies featured an adaptation of protein phylogenetic profile methods for investigating co-occurrence patterns in MCP operons, and led to an articulated classification of existing MCP pathways [13].

Here, we aim to characterize the molecular network of physical protein-protein interactions (PPIs) in a single MCP type, the Pdu system. In this case, strategies relying on genomic context have limited application due to the high similarity of the genomic patterns found for different proteins across the Pdu operons; essentially all of the MCP shell proteins and enzymes typically found in the Pdu operon are functionally linked according to genomic context, but only a subset engage in direct physical PPIs. Other computational strategies are therefore required to develop models for direct physical PPIs. Detailed sequence variations within protein families can be analyzed via phylogenetic tree-based approaches, and indeed methods based on mining of phylogenetic features have proven useful for predicting PPIs in multiple cases, as recently reviewed [65,66]. A non-exhaustive list of such methods includes the so-called *mirror tree* [67], or its variant the *tol-mirror* [68], which compares trees—one for each protein of interest—by computing the pairwise correlation of their underlying evolutionary distance matrices. Others explore the topological similarity of the trees, coined congruence by Vienne *et al*. [69]. All follow the co-evolution hypothesis, where interacting protein families are expected to exhibit similar phylogenetic trees with similar patterns of amino acid sequence divergence.

In this work, we seek to identify new PPIs in the Pdu MCP with a coevolution-based machine learning algorithm. Specifically, we approach the PPI prediction problem within a binary classification framework: from the pairwise comparison of phylogenetic trees, coevolution features can be computed and subsequently mined by a decision tree classifier, a concept earlier described in Craig and Lio [70]. A group of PPIs that have been experimentally characterized recently in the Pdu system constitute a set of known positives for use as a “gold standard” for training the classifier. In the first part of this work, we design and train a Random Forests classifier to identify pairwise interactions of Pdu gene products, and then propose a model of the Pdu interactome. Following this genomic-based model, we further analyze selected predictions of PPIs and their probable binding modes via three-dimensional protein-protein docking calculation. We then provide new experimental data to support one of the predicted interactions.

## Results

**Figure 2:**
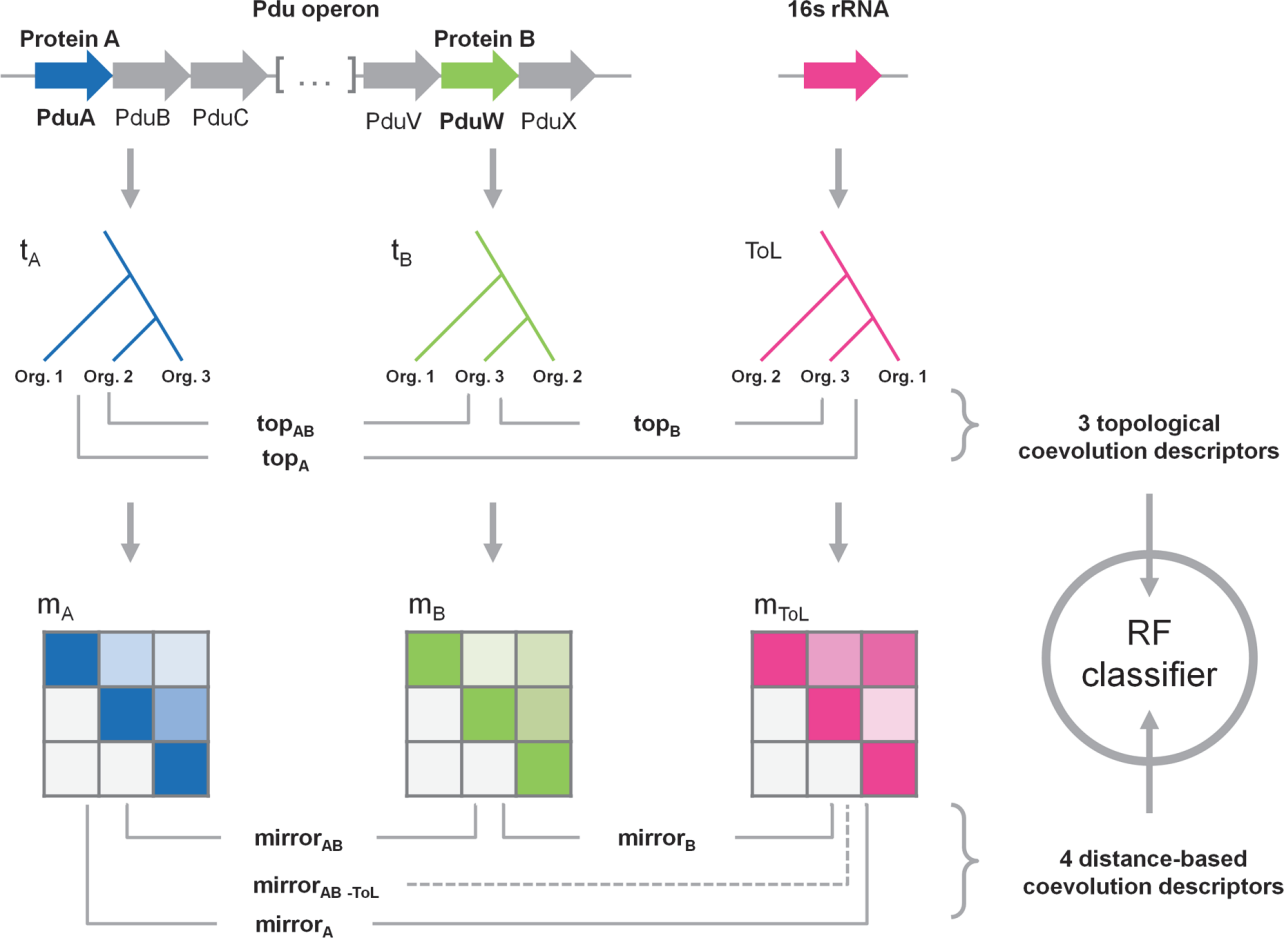
Description of the procedure for defining pairwise coevolution descriptors. Calculation of coevolution descriptors relies on the comparison of phylogenetic trees. For each given pair of Pdu gene products, three descriptors are extracted from a topological comparison of their respective phylogenetic trees (blue and green) and the Tree of Life (ToL, pink), while four other descriptors are calculated by comparing the distance matrices that underlie these trees. These seven descriptors are further combined into a vector for subsequent analysis by the RF classifier.

For each pair of Pdu gene products, we defined seven continuous-valued coevolution descriptors extracted from the pairwise comparison of their respective phylogenetic trees, and combined those seven values into a vector (Fig. 2). As an example, one of the seven descriptors is the linear correlation coefficient between two phylogenetic trees calculated by the *mirrortree* method. The other descriptors are variations on a similar theme (see Methods). Within this framework, and using experimental data on known interactions as a training set, we ran a binary classifier against these vectors of coevolution descriptors to identify positive PPIs.

### Predicting PPIs of the Pdu interactome

**Figure 3:**
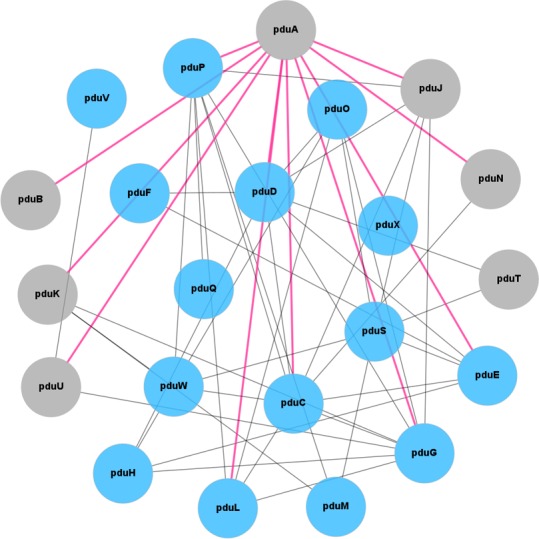
A model of the Pdu interactome. The Pdu PPI network, inferred from predictions made by the RF classifier in its analysis of coevolution descriptors. Individual Pdu gene products are represented as nodes. Enzymes are shown in light blue, while shell proteins are shown in gray; the shell proteins include several BMC type proteins and a single protein (PduN) from the BMV family presumed to be pentameric vertex proteins. Edges connecting two nodes correspond to predicted PPIs. The numerous PPIs emerging from the PduA node are highlighted in pink. It is not possible to fully convey the likely spatial relationships of all the proteins and enzymes (some of whose locations remain uncertain), but nodes for the shell proteins have been placed at the periphery of the layout to convey their outer locations.

We culled protein sequences from Pdu operons of 34 fully sequenced bacterial genomes, and collapsed them into 22 orthologous protein groups according to the canonical Pdu nomenclature [23]. For each of the 22 distinct protein families so identified, we inferred a phylogenetic tree from a multiple sequence alignment of its constitutive sequences. We refer to this as the ‘Pdu tree’ for that protein. Subsequently, for each pair of proteins seven co-evolution descriptors were computed from a comparison of their respective Pdu trees, following the general procedure depicted in Fig. 2. Pairwise combinations of the 22 orthologous protein groups resulted in 231 unique pairs that needed to be classified. For this purpose, we used a Random Forests classifier [71] exploring the seven descriptors, which after a training and cross-validation phase exhibited an area under the receiver-operator (ROC) curve of 0.75 (S1 Fig., suppl. Data), thereby demonstrating a reasonably good discriminative power. We also assessed whether similar classification performance could be obtained with fewer descriptors than the seven initially employed. We evaluated the discriminatory power of the descriptors individually by ranking their accuracies in the context of an unsupervised analysis (S2 Fig.). We found that the RF performs best when all seven of the descriptors are included in the classification analysis. Much of the signal can be recovered with just a few descriptors, but addition of subsequent descriptors does result in slight improvements in performance. When applied to the whole Pdu dataset, the classifier predicted a list of 109 positive PPIs along with their mean probabilities. To be conservative and increase the specificity of the classifier (even if at the expense of the sensitivity), we removed the putative PPIs with a probability less than 0.7, which reduced the final number of predicted PPIs to 51 (Suppl. data). From these results we modeled the Pdu interactome as a molecular network of 51 interactions and 22 nodes. The resulting network model is presented in Fig. 3.

An analysis of this model showed that 15 of the 16 experimentally characterized PPIs could still be retrieved under a high specificity criterion, and that they yielded the highest probabilities, confirming the robustness of the method. Furthermore, the missing positive interaction, PduK-PduT, was initially predicted as positive by the classifier, but did not pass our 0.7 threshold. One striking feature of this model is the absence of a PPI connecting the PduX node to the network (Fig. 3). PduX is an enzyme involved in *de novo* synthesis of coenzyme B_12_, an essential cofactor for enzymes of the Pdu pathway[72]. However, there is no evidence that PduX is directly associated with the MCP by any physical interaction [73]. Its tendency to occur within the Pdu operon (typically at the end) likely reflects an advantage of being under the influence of the Pdu promoter, rather than physical interaction with other MCP components. Two likely spurious findings involving interactions with PduF also appeared in our model, namely PduF-PduC and PduF-PduD. PduF is a propanediol/glycerol diffusion facilitator protein and is believed to be an integral membrane protein [74], making its physical presence in the MCP unlikely. Finally, after exclusion of the “gold standard” interactions and the suspected spurious predictions, the final dataset consisted of 36 predicted PPIs, with an average node connectivity of 4.8 partners, which can be loosely compared to results obtained with other interactome studies across whole cellular systems in yeast [75] or in cell junction complexes [76].

One intriguing observation is the hyperconnectivity of certain specific nodes, such as PduA (11 PPIs), PduC (9PPIs) and PduG (9 PPIs). The central position of the propanediol dehydratase large subunit PduC in the Pdu pathway makes it an essential piece of the interactome (Fig. 1B). Likewise, PduG is the large subunit of the diol dehydratase-reactivating factor, which works in tight coordination with the propanediol dehydratase (PduCDE). In a complex with PduH, PduG is believed to reactivate the dehydratase by exchanging its B_12_ cofactor, which becomes inactive during repeated catalytic cycling [73,77]. In our model, PduG was indeed predicted to interact with PduC and PduE but not with PduD. Although no structural information about this complex in *Salmonella* is available, crystal structures of highly similar homologs from *Klebsiella oxytoca* have been solved [22,78,79]. Studies with these same homologs demonstrated that the binding mechanism involved a subunit exchange between the dehydratase and the reactivase, where one PduH subunit is released from the reactivase and replaced by one PduD subunit [80].

Particularly notable in our interactome model is the number of PPIs in which PduA [73,81], one of the most abundant shell proteins in the Pdu MCP, is predicted to be involved. Presently, PduP is the only enzyme in the Pdu MCP whose binding to individual shell proteins has been characterized. It was revealed that PduP interacts via its N-terminal region with PduA and PduJ, another major shell protein that shares high sequence identity (83%) with PduA [36]. Other Pdu enzymes besides PduP are suspected to carry such N-terminal extensions [50], but their shell protein partners have not been identified yet [54,82]. Sequence analysis as well as spectroscopic experiments on the PduP N-terminal segment show that it has a strong propensity to fold into an alpha-helical structure [36,55]. Here, we hypothesize that these structural features and associated binding mechanism are not specific to the PduP case, but that PduA (or PduJ) likely serves as a central binding hub for different enzymes carrying N-terminal extensions. To pursue this particular set of interactions further, we generated atomic models of predicted PduA-enzyme-tail complexes by molecular docking and analyzed their predicted modes of binding.

Additionally, we analyzed the PduU-PduV case, the only PPI in which PduV was predicted. PduU was the first BMC shell protein from a non-carboxysome MCP whose three-dimensional structure was determined [44]. Its topology involves a circularly permuted BMC domain, and the existence of a six-stranded β-barrel capping the central pore of the hexamer makes it unique in the BMC protein family. Previous speculations about the peculiar beta barrel include a possible role in gating an unusually wide pore, but further data are lacking. Additionally, PduV is a Ras-like GTPase that has been implicated in MCP dynamics within the cell by Parsons *et al*. [82]. In this case, PduV is believed to reside outside the shell, as opposed to the other Pdu enzymes that appear to be sequestered in the MCP interior. To clarify how these two might interact, as predicted by our interactome model, we modeled the PduU-PduV complex with docking simulations and compared the result to control calculations involving non-interacting protein pairs.

### PduA: A universal hub for binding encapsulated enzymes

Of the 11 predicted interactions involving PduA, six include Pdu enzymes, namely PduC, PduD, PduE, PduG, PduP, and PduL (Fig. 3). As noted above, one of these interactions (PduA-PduP) has been demonstrated experimentally. Here we investigated whether enzymatic partners in addition to PduP are also able to bind PduA via terminal peptides, by attempting to model their presumptive binding modes computationally (see Methods).

As a first step, we searched for possible terminal peptidic extensions in the sequences of these six Pdu enzymes. Prediction of these extensions was done according to the method developed in Fan *et al*. [50]. The central idea is that enzymes that are targeted to the MCP exhibit extensions at their termini that are absent from homologous versions of the same enzyme that are not part of an MCP system. It is notable that among the six enzymes that are predicted by our model to bind to shell protein PduA (or its close homolog PduJ), our computational analysis indicates that five carry recognizable sequence extensions (PduC,D,E,P,L), (as reported in [50] and [54]). In contrast, none of the 15 enzymes that do not have predicted interactions with PduA (or PduJ) exhibit recognizable terminal sequence extensions. Sequence comparisons between the N-terminal peptide tails did not reveal strong similarities (less than 30% identity overall). However, *ab initio* predictions of their structures consistently modeled them as amphipathic α-helices. Experimental studies have already investigated the possible targeting of some of the Pdu enzymes; targeting by the N-terminal tail of PduP was noted above [50]. In the case of PduD, experiments showed that its N-terminal peptide can be used as a targeting signal, but there was no evidence that it would fold identically to the PduP peptide or that its interaction would be with PduA [54]. In these same studies, PduE was implicated as having such terminal extensions, but fusion of GFP to its respective peptides did not provide clear evidence for targeting. In the case of PduC, Parsons, et al. showed that that enzyme could direct other proteins to the MCP when fused genetically, though the presence of a terminal tail on PduC was not indicated [81]. Despite the mixed findings on terminal targeting peptides on different enzymes in different experimental protocols, the presence on several of the Pdu enzymes in our bioinformatics analysis of extended termini with predicted alpha-helical propensities, and the prediction here of interactions between those enzymes and the PduA shell protein, supported the idea that some of these peptides likely recognize the interior surface of the shell using similar binding modes.

Since it was demonstrated that the targeting of PduP is mediated mostly by its terminal peptide segment [50], we sought to characterize the binding mode of the various implicated enzymes by docking their N-terminal peptides onto the hexameric structure of the PduA shell protein; 18-amino acid terminal segments were used in all cases. The benefits of using a model of the terminal peptide instead of a complete protein are twofold: (1) to avoid spurious modeling of full-length proteins in the absence of close structural homologs, and (2) to substantially reduce the size of the search space to be explored by the docking algorithm. In earlier work we proposed a model of the PduP N-terminal extension bound to the concave face of a PduA hexamer (proposed to be inward facing) [35]. However, this model was generated with a rigid-body approach, where the PduP peptide had only flexible side chains. Here we push further the flexibility limits of the docking simulation by additionally allowing conformational degrees of freedom for the peptide backbone. To do so, we employed a two-stage docking approach: a rough search by Autodock Vina [83] of the binding site in the PduA hexamer with a rigid helical model of the peptide, followed by a second docking phase with the FlexPepDock protocol from the Rosetta suite [84]. In this second step, the peptide is placed in its start position according to Vina’s predictions; it is then simultaneously refolded and docked over the surface of the receptor. We applied this approach to the five identified PPIs and to a control case involving the N-terminal sequence from PduQ, an aldehyde dehydrogenase from the Pdu pathway that has no obvious targeting signal. In addition, the five peptides were alternatively docked on both faces of the PduA hexamer, with the expectation that meaningful results would have peptides docking to only one side of the PduA shell protein.

**Figure 4:**
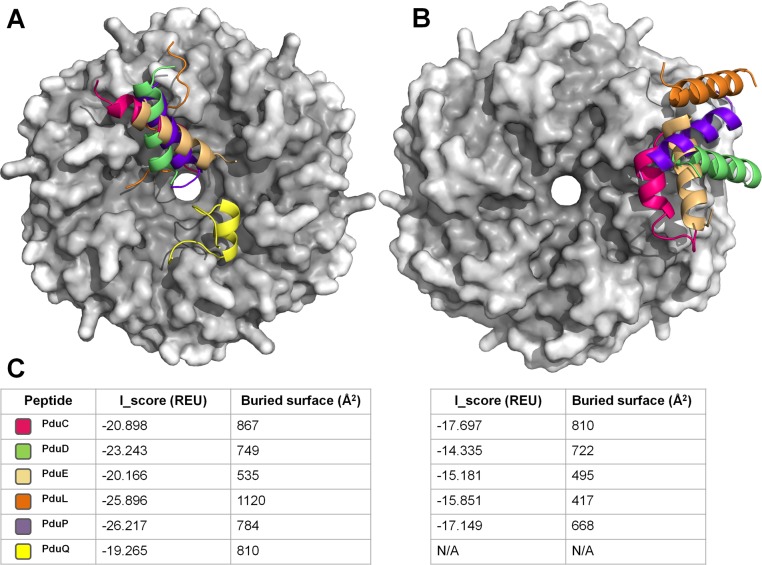
Models of N-terminal peptide extensions from different enzymes docked onto a PduA hexamer. All the models were aligned and overlaid using the PduA structure as guide. (A) Six N-terminal peptides are docked on the concave (presumptively luminal) face of the PduA hexamer. Four of the five identified earlier as probable targeting sequences (PduC, PduD, PduE, PduP) were folded into α-helices by the flexible docking procedure (see text and Methods) and were docked in the same cleft on the PduA surface. The tail of PduL adopted a less regular conformation during the simulation. The tail from PduQ, which was not predicted to act as a targeting sequence and thereby serves as a control, exhibits an apparently spurious binding mode. To convey depth, the surface of PduA is shaded according to diffusion accessibility [106]. (B) The five targeting peptides, when docked onto the other (flat) face of the PduA shell protein, were found scattered across the surface in arrangements exhibiting poorer interaction interfaces. (C) Binding statistics are reported for all the docking simulations. In all cases, both the predicted energy score (in Rosetta Energy Units) and the buried surface at the interface yielded better values when peptides were docked onto PduA’s concave side. Because the shell protein hexamer is 6-fold symmetric, in all cases the solutions were rotated by multiples of 60° around the axis of symmetry to allow internal consistency.

**Figure 5:**
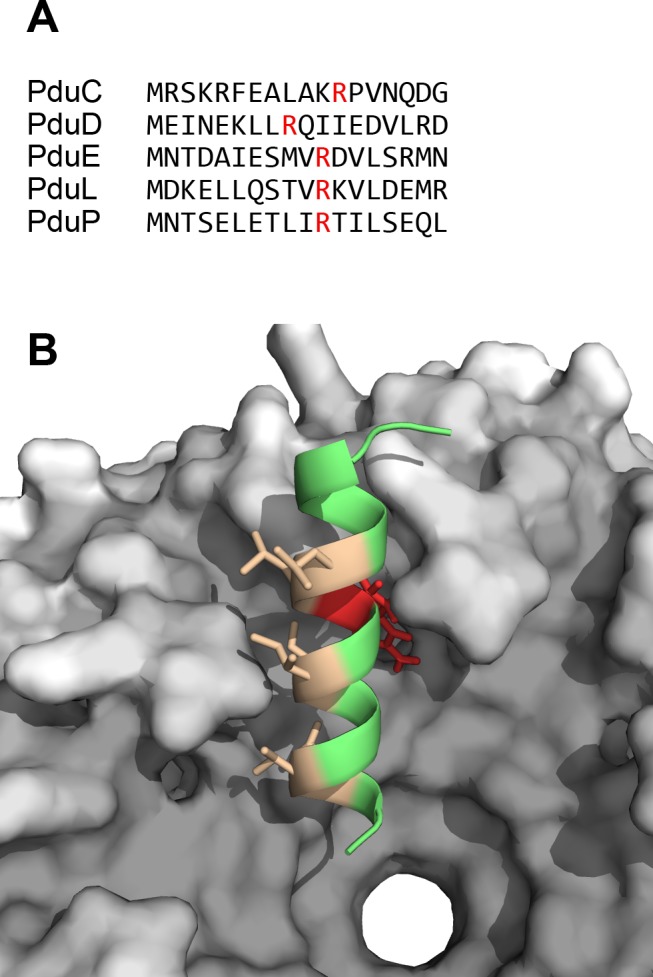
N-terminal extension sequences and atomic details of the PduD peptide docked onto a PduA hexamer. (A) Sequences of the five N-terminal extensions proposed to be acting as targeting peptides. An arginine is recurrently found near the center of the peptide (red). (B) The hydrophobic surface (in beige) of the PduD N-terminal tail peptide is predicted to interact with the C-terminal tail of PduA. A central arginine (in red), which is found in all of the N-terminal peptides predicted to dock in the cleft, is consistently oriented to make interactions with a glutamate in the BMC domain.

Results of the peptide docking simulations are overlaid in Fig. 4 along with their energy scores and their buried surface areas. Remarkably, when docked onto the concave (presumptively luminal) face of PduA, all five peptides were predicted to bind the same binding cleft formed by the C-terminal segments of two adjacent PduA monomers in the hexamer (Fig. 4A). Moreover, with the exception of PduL, FlexPepDock automatically folded the peptides into well-defined α-helical structures. In the case of PduA-PduP, the model is similar to the one initially proposed in Yeates *et al*. [35], with a slight rotation and translation inside the cleft. Interestingly, the different peptides occupy the common binding cleft of PduA in different orientations: PduP and PduE have their N-termini pointing toward the pore, whereas PduC and PduD are docked in the opposite direction. The PduL peptide was also predicted to bind roughly the same region, but the flexible docking protocol did not automatically fold that peptide into a well-ordered alpha helix, leaving the veracity of the predicted binding mode in question in the case of PduL. In their computationally predicted bound configurations, most of the polar residues of the peptides are exposed to the solvent. A notable exception is an arginine recurrently found towards the center of each peptide, which is in all cases buried in the predicted interface and poised to form a salt-bridge with glutamate (E36) of either one of the two monomers constituting the binding cleft (Fig. 5A). The hydrophobic residues are oriented to interact with the C-terminal segment of PduA (Fig. 5B).

Various other docking calculations served as computational controls. In contrast to the results obtained for docking to the concave surface of the shell protein, docking of the peptides on the other (flat) side of PduA showed no consistent or compelling modes of binding. Those peptide models are instead scattered over the hexamer surface (Fig. 4B). Moreover, comparison of the energy scores and buried surface areas in both docking cases shows that the peptides have a significantly better fit to the concave surface (Fig. 4C). Another control consisted of docking the N-terminal 17 residues from PduQ (which was not predicted to have a targeting tail) following the same protocol. In the docking simulation the PduQ peptide partially folds into an α-helix, but does not seem to bind intimately in the canonical cleft (Fig. 4A). An additional calculation involved the docking of N-terminal peptides onto a layer of three PduA hexamers packed side-by-side, to verify that potential binding modes at the interfaces between hexamers were not overlooked. This simulation exhibited similar binding modes to those found with a single PduA hexamer. Overall, these computational predictions and control calculations support the hypothesis that the interior surface of PduA serves as a hub for binding multiple enzymes with terminal extensions. The findings are largely consistent with previous experimental data, while painting a more detailed picture of how interior enzymes in the Pdu MCP interact with PduA, as predicted by our coevolution analysis.

### A predicted PduU-PduV complex

**Figure 6:**
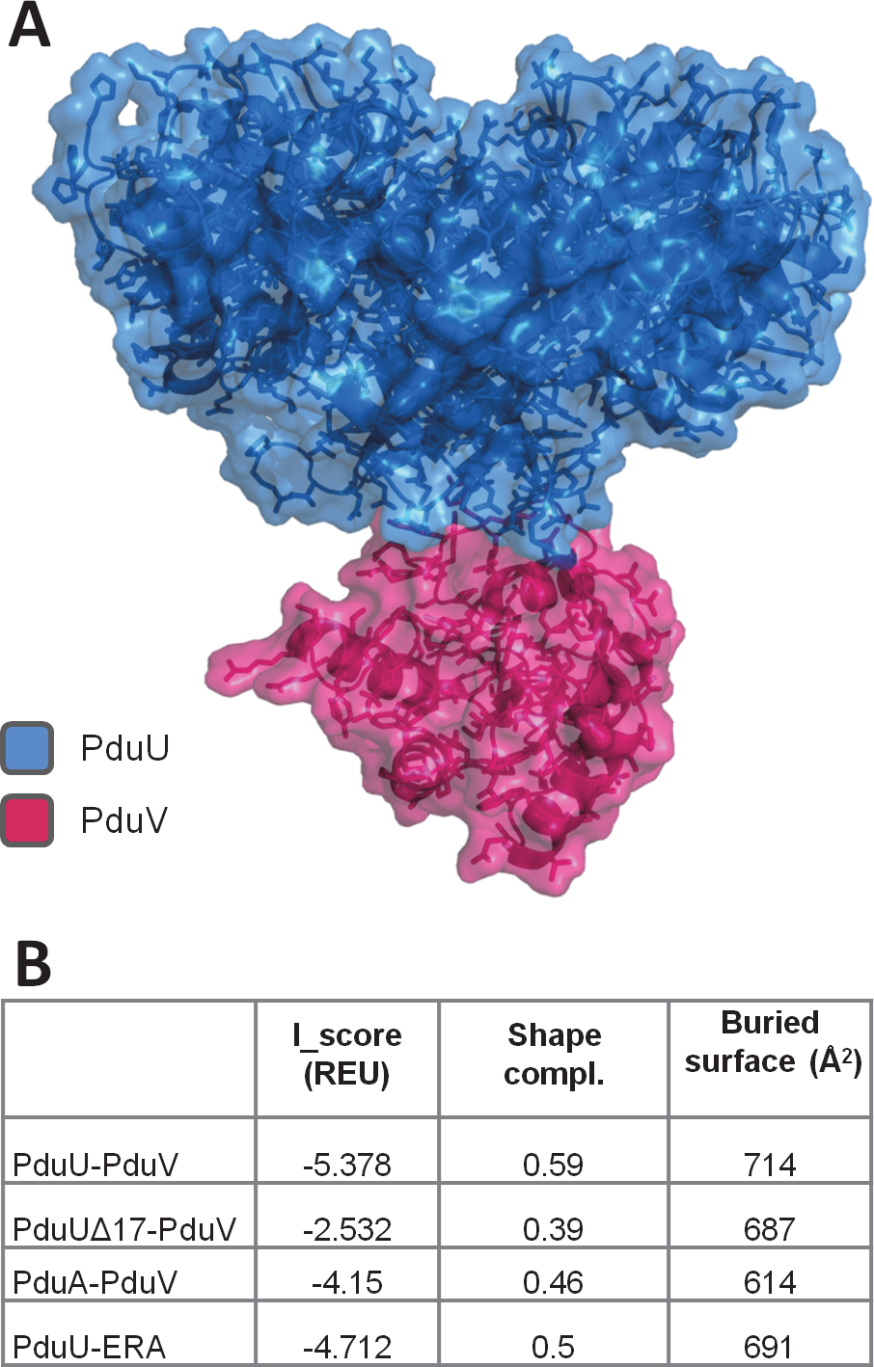
Model of PduV docked onto a PduU hexamer. (A) Docking calculations predict that the N-terminal region of PduV binds the PduU β-barrel that protrudes from the conserved BMC domain. Binding statistics for the PduU-PduV docking and three control simulations are reported in a separate table (B). Those latter, which included the docking of PduU to a non-cognate GTPase homolog of PduV (labeled PduU-ERA), a truncated version of PduU lacking the beta-barrel docking to PduV (labeled PduU Δ17-PduV), and PduV docking to PduA instead of PduU (labeled PduA-PduV), all had substantially worse binding statistics than the PduU-PduV model.

As an initial step in modeling a possible interaction between PduU and PduV, which was predicted by the coevolution analysis, a homology model had to be constructed for PduV. The PduV model was then docked into the crystal structure of the PduU hexamer using RosettaDock [85] (see Methods). As a control, we ran two docking simulations under identical conditions on cases involving either PduU or PduV and non-interacting molecules: PduA-PduV, and PduU-ERA (the homologous GTPase used as the template for modeling of PduV). A model of the PduU-PduV complex is proposed in Fig. 6A, along with statistics from the different docking simulations (Fig. 6B). Compared to the two controls, the predicted interface between PduU and PduV achieved a better Rosetta energy score. Likewise, the PduU-PduV complex featured a better shape complementarity and larger buried surface than the controls. In this model, PduV is sitting on the axis formed by the PduU beta-barrel; this PduU protuberance is exclusively contributing to the interface and precludes any interaction between PduV and the main BMC domain of PduU. Most of the interaction surface on PduV is formed by the N-terminal region spanning residue 13 to 35. This is consistent with preliminary results from Parsons *et al*., where the first 42 amino acids from PduV were demonstrated to play a crucial role in PduV targeting to the MCP [82]. As a final control calculation, we investigated the binding mode of PduV after deleting the 17 N-terminal residues forming the β-barrel in the PduU hexamer. Here again, the model yielded worse interaction statistics than for the full-size PduU-PduV complex, supporting the model in which the β-barrel of PduU plays a crucial role in the interaction with PduV (Fig. 6B).

### Experimental confirmation of a PduU-PduV interaction

Preliminary experimental assays were carried out on the PduU-PduV pair in parallel with our computational analysis. The BacterioMatch II two-hybrid system was used to test for interactions between these two proteins. In this system, a reporter strain is co-transformed with appropriate target and bait fusion genes. A protein-protein interaction between the target and bait activates the transcription of *HIS3*, an essential gene for histidine biosynthesis [86], thereby increasing the expression of the *HIS3* product to levels that are sufficient to allow growth on a selective medium lacking histidine and to overcome the effect of 3-amino-1,2,4-triazole (3-AT), a competitive inhibitor of the His3 enzyme. If a large number of colonies are obtained following co-transformation, an interaction between the target and bait proteins is indicated. When PduU and PduV were tested, the number of colonies obtained following co-transformation was comparable to that of a positive control with bait and prey proteins (LGF2 and Gal11^P^) that are known to strongly interact (Table 1). Results showed that PduU and PduV also interacted in reciprocal tests where their roles as bait and prey were reversed (Table 1). Negative controls showed that PduU or PduV alone did not confer 3-AT resistance (Table 1). The positive result with the UV pair was confirmed by streptomycin resistance of co-transformed *E. coli* which requires expression of a second reporter gene, *aadA*. This experimental confirmation of a PduU-PduV interaction supports the Pdu MCP interactome model developed in the first (coevolution analysis) part of our work, while the docking calculations reveal a plausible mode of binding between those proteins.

**Table 1:** Two hybrid assay to test the interaction between PduU and PduV.

Pairs tested	NS^a^	NS 1:100^b^	S^c^
pBT-*LGF2*,pTRG-*Gal11^P^*	TNTC^d^	1000 ± 87	1000 ± 72
pBT-*pduU*,pTRG	TNTC	11 ± 3	0 ± 0
pBT-*pduV*,pTRG	TNTC	19 ± 10	0 ± 0
pBT, pTRG-*pduU*	TNTC	167 ± 21	0 ± 0
pBT, pTRG-*pduV*	TNTC	740 ± 75	7 ± 3
pBT-*pduU*, pTRG-*pduV*	TNTC	1000 ± 66	600 ± 12
pBT-*pduV*, pTRG-*pduU*	TNTC	840 ± 79	TNTC

Different combinations of bait(pBT) and prey (PTRG) genotypes are tested. The LGF2 and Gal11^p^ pair serves as a positive control. Tests with each pair were repeated three times and the numbers shown are the mean±1 standard deviation.

^a^cfu obtained on nonselective (NS) screening medium (no 3-AT) plates.

^b^cfu obtained on nonselective screening medium plates with 1:100 diluted cells

^c^cfu obtained on selective (S) screening medium (with 3-AT) plates.

^d^too numerous to count

## Discussion

Proteins rarely carry out biological processes on their own. Instead, they typically participate with other proteins in the context of larger interaction networks. This is especially true for MCPs, where encapsulated pathways require coordination and spatial organization of their numerous components, from shell proteins to enzymes. Though structural studies of individual MCP components have paved the way to a better understanding of their assembly mechanism, a full comprehension of such metabolic systems requires investigation of their PPI networks. Unfortunately, experimental data for MCP protein complexes are still sparse, leading us to turn to predictive methods. Here, we used coevolution calculations and a binary classifier to predict pairwise PPIs in the Pdu MCP, and proposed a model of its interactome. Approaches using binary classifiers for coevolution-based PPI predictions have been developed by others. Comparable approaches have been successfully applied to *E.coli* [87], and to the human genome [88]. Interpreting such networks is not a trivial task, considering that such methods are predictive in nature and can therefore include spurious predictions of PPIs or, *a contrario*, miss true interactions. Additionally, these methods cannot always distinguish direct (i.e. physical binding) and indirect (functional) correlations, a recurrent problem in coevolution studies that is illustrated here by the integration of PduF in our network. In order to mitigate the deficiencies of the computational methods we employed, a conservative approach was taken by considering only those predicted interactions that had the highest probability (p≥0.7). These cases were largely consistent with existing experimental data, where they were available. An example of a positive result is the agreement between our predictions and structural data relating to the reactivation mechanism of the diol dehydratase [80].

Extending on our predicted interactome model, we focused further analyses on PPIs emanating from the PduA shell protein node and involving Pdu enzymes (Fig. 3). Of these PPIs, five where identified as presenting an N-terminal extension, a characteristic of lumen-targeted enzymes. These N-terminal peptides, when docked onto a PduA hexamer, consistently bound the same cleft on the concave surface of the hexamer. Likewise, most of them folded into amphipathic α-helical structures, their hydrophobic faces oriented towards the C-terminal tail of the PduA shell protein, a region somewhat less conserved than the main BMC domain. These atomic details are depicted in Fig. 5, where for example the PduA-PduD case is more clearly pictured. These results are consistent with experimental studies by Fan *et al*., which demonstrated the necessity of the PduA C-terminal helix in PduP binding and the role of hydrophobic residues in that interaction [36]. An exceptional case during these docking simulations was the PduL peptide, which did not fold into an amphipathic helix. With regard to our inability to obtain a robust docking result with a PduL peptide, it is notable that the interior vs exterior location of PduL remains unclear in current models of the Pdu MCP. If it is interior, its enzymatic reaction (depicted in Fig. 1B) could internally recycle the coenzyme A used by PduP for the conversion of propionaldehyde to propionyl-coA. Indeed, a similar mechanism is used for HS-CoA recycling by the Eut MCP [89] and has been demonstrated for NAD^+^ recycling by PduQ [90]

The results of our docking studies are of particular significance for the issue of sidedness of the MCP shell—i.e. which side of the shell proteins faces inward vs. outward. Previous arguments have suggested that the concave side of the shell protein faces into the MCP lumen [35,37,38]. Mutagenesis experiments by Fan *et al*. on the PduA C-terminal helix support that assignment [36]. In our present docking study, the consistent binding of the targeting peptides onto the concave side of the PduA hexamer, and the consistently better interface statistics compared to docking on the other side, strongly corroborate this idea.

PduA and PduJ, two highly similar paralogs of the BMC shell protein, are the two most abundant shell proteins after PduBB’ in the Pdu system. As a consequence, they are suspected to play a critical structural role [73]. Indeed, while deletions of *pduK, pduT* or *pduU* do not affect the formation of the MCP, *pduA* mutations produce disrupted or enlarged shells [82,91]. Pull-down assays confirmed this architectural importance, where PduA was shown to interact with multiple other shell proteins [82]. Here we suggest that in addition to its transport and structural roles, PduA likely serves as a universal hub for a clique of cargo enzymes, attaching them to the shell via their N-terminal extensions. The highly similar shell protein PduJ is also predicted to interact with four of the same six enzymes associated with PduA. A possible interpretation is that the same clique of enzymes is able to bind both PduA and PduJ, some pairs being more thermodynamically favored than others. Another explanation would be that these PPIs are in fact exclusive, but that our approach is not sensitive enough to discriminate PPIs involving close homologs. Note that the absence of an available structure for PduJ prevented a comparison by computational docking. Whether PduA and PduJ have similar or different affinities for various enzymatic partners will require further investigations, including experimental studies.

Attributing a special functional role to PduA (or PduJ) is consistent with the view that, though the multiple paralogous shell proteins in the MCP share a canonical BMC structure, each shell protein variant fulfills a specific task. For instance, tandem BMC proteins such as EutL are proposed to regulate the transport of metabolites via conformational changes and a gated pore [38,40,92,93]. The recent crystal structure of PduB, a EutL homolog, presents a view of a tandem domain shell protein from the Pdu system in a closed conformation [46]. Another apparently specialized shell protein is PduT, a tandem BMC domain shell protein that is suspected to bind an iron sulfur cluster in its central pore [39,45].

In this portrait of the Pdu family, the role of PduU remains to be elucidated. Here, we aimed to bring new clues by investigating the intriguing PduU-PduV case. Indeed, PduV is also poorly characterized compared to other Pdu components. Furthermore, from our predictions, PduV was the only enzyme exclusively interacting with a shell protein. The diverse docking simulations involving PduU and PduV all agreed with the existence of such a PPI, and predicted the N-terminal region of PduV binds directly to the PduU beta-barrel, consistent with recent experimental data on the importance of the N-terminus of PduV [82]. These predictions, coupled to our preliminary experimental data on a PduU-PduV interaction, fill a gap in understanding the role of the unique β-barrel in PduU.

To conclude, the present study brings further insights into the organization of the Pdu MCP, and constitutes the first systematic computational effort to describe an MCP interaction network. The basis of this work is predictive, but we have investigated one of the predicted interactions experimentally as part of this investigation, with a positive result. Further experimental studies will be required to more fully evaluate the interactome model developed here. Application of the same approach to other types of characterized MCPs might be of equal interest and could reveal similar insights.

## Materials and Methods

### Collection of Pdu operons and construction of phylogenetic trees

Protein orthologs were collected from 34 bacterial genomes in the KEGG database [94] and collapsed among the 22 types of MCP proteins known to be associated with the Pdu system: *pduABCDEFGHJKLMNOPQSTUVWX* (Suppl. Data). Incomplete or erroneous annotations of the Pdu gene products were corrected after sequence comparison with the Pdu operon from *Salmonella enterica* LT2, the best-characterized strain in terms of Pdu MCP.

For each ortholog group, its corresponding protein sequences were aligned with MUSCLE [95]. The multiple sequence alignments were subsequently input in PhyML [96] for the construction of phylogenetic trees using the Maximum Likelihood method. Since some of the co-evolution descriptors also involve the Tree of Life of the 34 genomes studied, sequences of their respective 16S ribosomal RNA were submitted to similar treatment. For amino acid and nucleotide-based tree construction in PhyML, we used the LG [97] and HKY85 [98] substitution matrices, respectively. Additionally, distance matrices were calculated for each tree, where the distance between two leaves corresponds to the sum of the branch lengths separating them.

### Dataset construction

Seven coevolution descriptors measuring the pairwise tree similarities have been defined. Of these, four are based on pairwise comparison of the distance matrices, as defined in the *mirrortree* approach, while three others reflect topological similarities (Fig. 2). In the former class of descriptors, the metrics correspond to the linear correlation coefficient between the two matrices in consideration, while in the latter, it involves the congruence index *I_cong_* as defined in Vienne *et al* [69]. Noteworthy is the fact that comparing two trees can be subject to artefacts and lead in some cases to spurious correlations if speciation events are not taken in account. For this reason, some of these descriptors involve comparisons of the individual proteins to the Tree of Life. Let A and B be the two MCP ortholog groups, m_A_ and m_B_ their respective matrices, t_A_ and t_B_ their trees, and ToL the Tree of Life of the 34 genomes. The parameter mirror_AB_ is the correlation between m_A_ and m_B_, mirror_A_ is between m_A_ and ToL, and mirror_B_ is between m_B_ and ToL. The fourth descriptor, mirror_AB-ToL_, involves an adaptation of the mirror tree, also known as tol-mirror [68], which measures the correlation between m_A_ and m_B_ after removing the background similarity inherent to speciation events in the ToL. Since distances in the ToL are computed from a nucleotide-based substitution matrix, the distances in the ToL matrix have to be rescaled as proposed in [68] for proper comparison with the protein-based distance matrices.

Topological descriptors are derived from the *I_cong_* index, defined as the probability that the Maximum Agreement Subtree (MAST) between two trees is arising by chance. Along the same idea, topological similarities were computed between tree A and ToL, tree B and ToL, and finally A and B ( top_A_,top_B_,top_AB_).

### Binary classifier

We implemented a Random Forests (RF) classifier [71] from the Weka Library in Java [99]. Two classes were defined: *pos* for an interacting protein group pair and *neg* for those not interacting. Each of the ortholog group pairs sees its input vector of seven coevolution descriptors evaluated by the RF classifier. To classify a pair, its input vector is run through each decision tree of the forest and sees its mean probability attributed. The mean probability threshold for distinguishing the *pos* from the *neg* cases was set to 0.5, where a probability ≥ 0.5 will classify the pair as *pos*.

### Gold standard and cross validation

The dataset used for training the RF classifier—the “gold standard”—was derived from experimental data found in the literature on the Pdu MCP. Manual mining of this data led to a total of 40 pairs of Pdu proteins whose physical interactions (or lack of interaction in many cases) could be verified experimentally via binding assays [36,82,90,100,101], complementation and expression studies [22] or crystallographic data [79]. An example of a verified non-interaction would be a direct binding experiment in which one protein component of a candidate pair failed to pull down the other. Among these, 16 are actual PPIs while the remaining 24 are non-interacting pairs. Of the 16 PPIs, 4, 6 and 6 pairs fall within the categories of: shell-enzyme (S-E) interactions, shell-shell (S-S) interactions and enzyme-enzyme (E-E) interactions, respectively. Likewise, the non-interacting pairs include 12 S-E, 12 S-S and zero E-E interactions. Each of these cases was assigned a class according to the rules defined earlier. The reported AUC value (0.75) for the classifier was calculated after a 10-fold cross validation. In parallel, we also carried out a 5 -fold cross validation that yielded a comparable AUC (0.73).

### Interactome representation

The interactome was pictured as an undirected graph with the igraph library in R [102]. Nodes and edges were computed with a Fruchterman-Reingold layout [103].

### Modeling of interacting partners with no structural information

While the structures of PduA and PduU are available in the PDB [104], structural information on the specific enzymes believed to interact with the shell proteins is limited. A recent NMR structure of the PduP tail showed an alpha helical structure, consistent with sequence-based predictions. Similar data are not available for the tails of the other enzymes of interest. We elected to assume as little as possible about the various tail structures and to model *ab initio* the first 18 residues of each enzyme with the PEP-FOLD server [105].

PduV was not presumed or predicted to bind by way of a terminal extension, so a model of that intact enzyme fold was required for docking analysis. The structure of PduV is presently unknown. Therefore, to enable computational docking, we built a homology model with I-TASSER [106] by threading the sequence of PduV onto two structural templates from the PDB (3IEV_A and 3R9W_A). The final model achieved a TM-score of 0.76, which is reasonable for further investigation by docking simulations [107].

### Docking simulations

For protein-peptide docking, our approach relied mainly on the Rosetta-based protocol FlexPepDock [84]. Its ability to simultaneously fold and dock allows full flexibility of the peptide. However, FlexPepDock sees its accuracy decrease when the starting peptide conformation has an RMSD higher than 5.5 Å compared to the native structure. Mindful of this constraint, we designed a two-step method for docking the N-terminal enzymatic peptides onto the PduA hexamer. The first stage is a coarse-grained search of the approximate binding mode by AutoDock Vina [83]. This model is subsequently refined by an *ab initio* FlexPepDock run, where the Vina model is used as an input coordinates file. Vina has been designed for small molecule docking, which allows a ligand flexibility up to 32 rotatable bonds only, a limit not existing in FlexPepDock. However, it can still be used efficiently when medium-sized ligands like peptides are treated as semi-rigid for predicting an approximate binding region. File preparation for AutoDock Vina included a configuration file specifying an exhaustiveness of 10 and a 27000 Å^3^ grid box encompassing the surface of the hexamer and centered on the pore. Coordinate files in PDBQT format were generated from the PduA crystal structure and the PEP-FOLD models of each peptide. For the peptides, rotatable bonds were defined for the side chains while Kollman United Atom charges were assigned to both the hexamer and the peptides. The pose computed by Vina with the lowest energy score was subsequently considered as the starting point for FlexPepDock. In this second stage, we ran 10000 simulations where the peptide was completely refolded and docked into the PduA hexamer. After ranking the 10 000 poses by lowest Rosetta energy, the top 500 poses were collapsed into clusters for which the internal RMSD was less than 2.5 Å. Finally, we picked the definitive model as the one with the lowest energy among the two most populated clusters.

For the PduU-PduV case, we used a standard RosettaDock protocol where the input included coordinates of both partners in their unbound state, typically those from the PduU hexamer and and the PduV homology model. The number of simulations, the ranking, clustering and selecting methods were identical to the FlexPepDock procedure, while the allowed flexibility in this case is limited to the side chains.

### Two-hybrid assay on the PduU-PduV pair

To test for interactions between PduU and PduV, the BacterioMatch II two-hybrid system (Agilent technologies) was used according to the manufacturer’s instructions with the following modification: co-transformation was carried out by using 30 ng each of the bait and prey vector. To construct the needed plasmids, *pduU* and *pduV* DNA sequences were amplified by PCR and then restricted and ligated into pBT for expression as fusions with the λcI protein, and into pTRG for expression as fusions with the RNAPα protein. Ligation reactions were used to transform *E. coli* XL1-Blue MRF’. Plasmid DNA was purified using a Qiagen mini prep kit, and all clones were verified by DNA sequencing. Self-activation by each recombinant bait and prey was tested before the two-hybrid interaction assays to determine if the bait or prey was capable of activating the reporter cassette on its own. Determination of protein-protein interaction was carried out by co-transforming BacterioMatch II validation reporter competent cells using recombinant bait and target.

## Supporting Information

S1 Fig Receiver Operating Characteristic(ROC) curves for the RF classifier with different combination of co-evolution descriptors. The quality of the RF classifier was assessed for three different combinations of coevolution descriptors: One combines the descriptors based on direct relationships between two proteins (A and B in Fig. 1) and exhibits an Area under the ROC Curve(AUC) of 0.47(green). The second scenario, which combines only the descriptors based on comparison between the Tree Of Life and one of the protein (A or B) obtains an AUC of 0.67(purple). A third case that uses all descriptors yields the best performance with an AUC of 0.75 (blue).
(TIF)

S2 Fig Assessment of the classifier performance using incremental combinations of coevolution descriptors. AUC values were calculated after running the RF classifier with different incremental combinations of the descriptors, starting from the most accurate and adding the next best one at a time. Here again the classifier yields the best performance when combining all the descriptors.
(TIF)

S1 Dataset List of probabilities of protein-protein interactions in the Pdu MCP. Protein-Protein interactions predicted by the RF classifier along with their respective mean probabilities (PPI in bold had P >0.7 and were used for the construction of the Pdu interaction network pictured in Fig. 3).
(DOCX)

S2 Dataset KEGG ID of the genes encoded within the Pdu operons analyzed in this study. (DOCX)
