# Supplementary figures and images for: Exploring Bacterial Organelle Interactomes: A Model of the Protein-Protein Interaction Network in the Pdu Microcompartment
Source: PLoS Comput Biol. 2015 Feb 3;11(2):e1004067. doi: 10.1371/journal.pcbi.1004067 (PMC4315436; doi:10.1371/journal.pcbi.1004067)

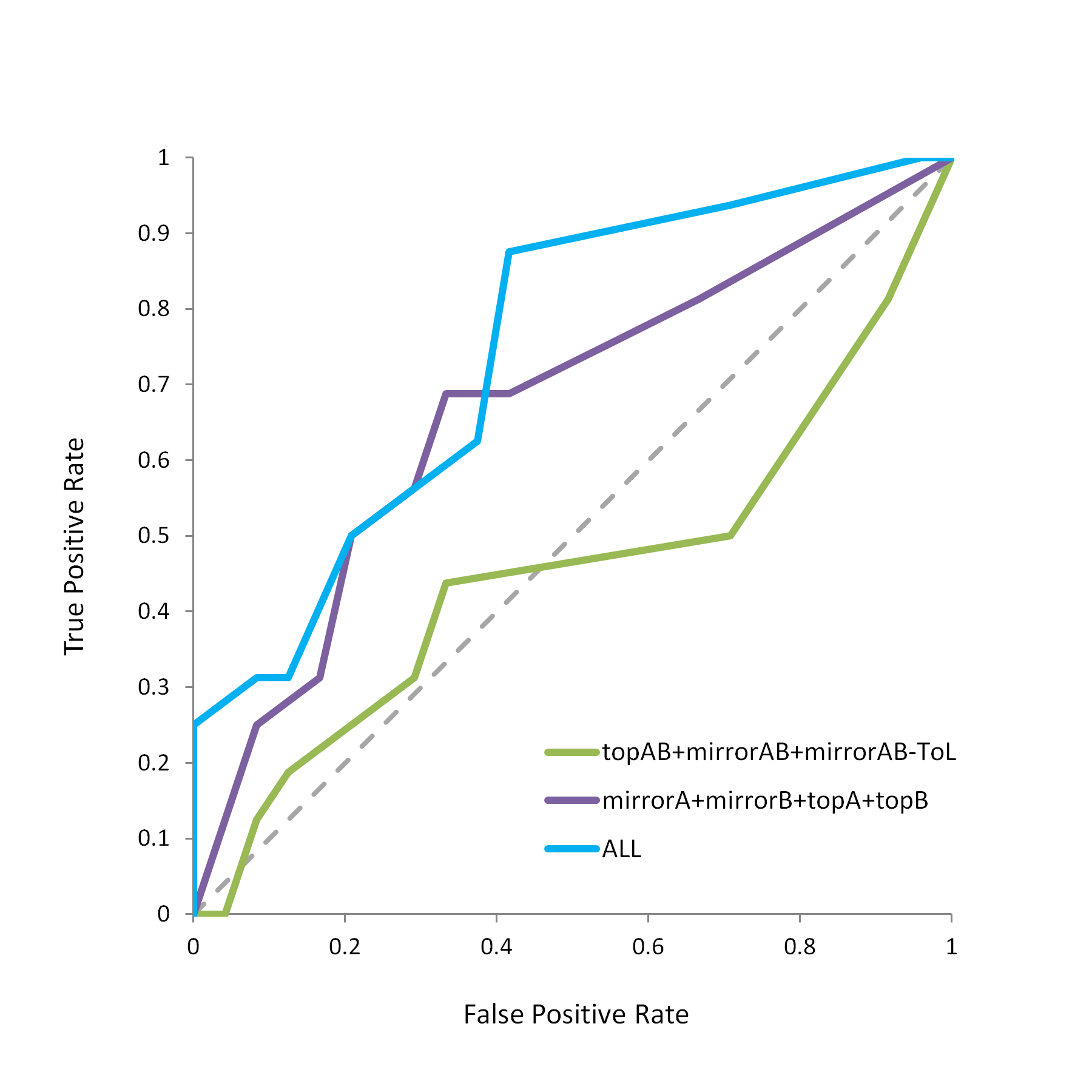

Supplement: S1 Fig — The quality of the RF classifier was assessed for three different combinations of coevolution descriptors: One combines the descriptors based on direct relationships between two proteins (A and B in Fig. 1) and exhibits an Area under the ROC Curve(AUC) of 0.47(green). The second scenario, which combines only the descriptors based on comparison between the Tree Of Life and one of the protein (A or B) obtains an AUC of 0.67(purple). A third case that uses all descriptors yields the best performance with an AUC of 0.75 (blue). (TIF) [file pcbi.1004067.s001.tif]

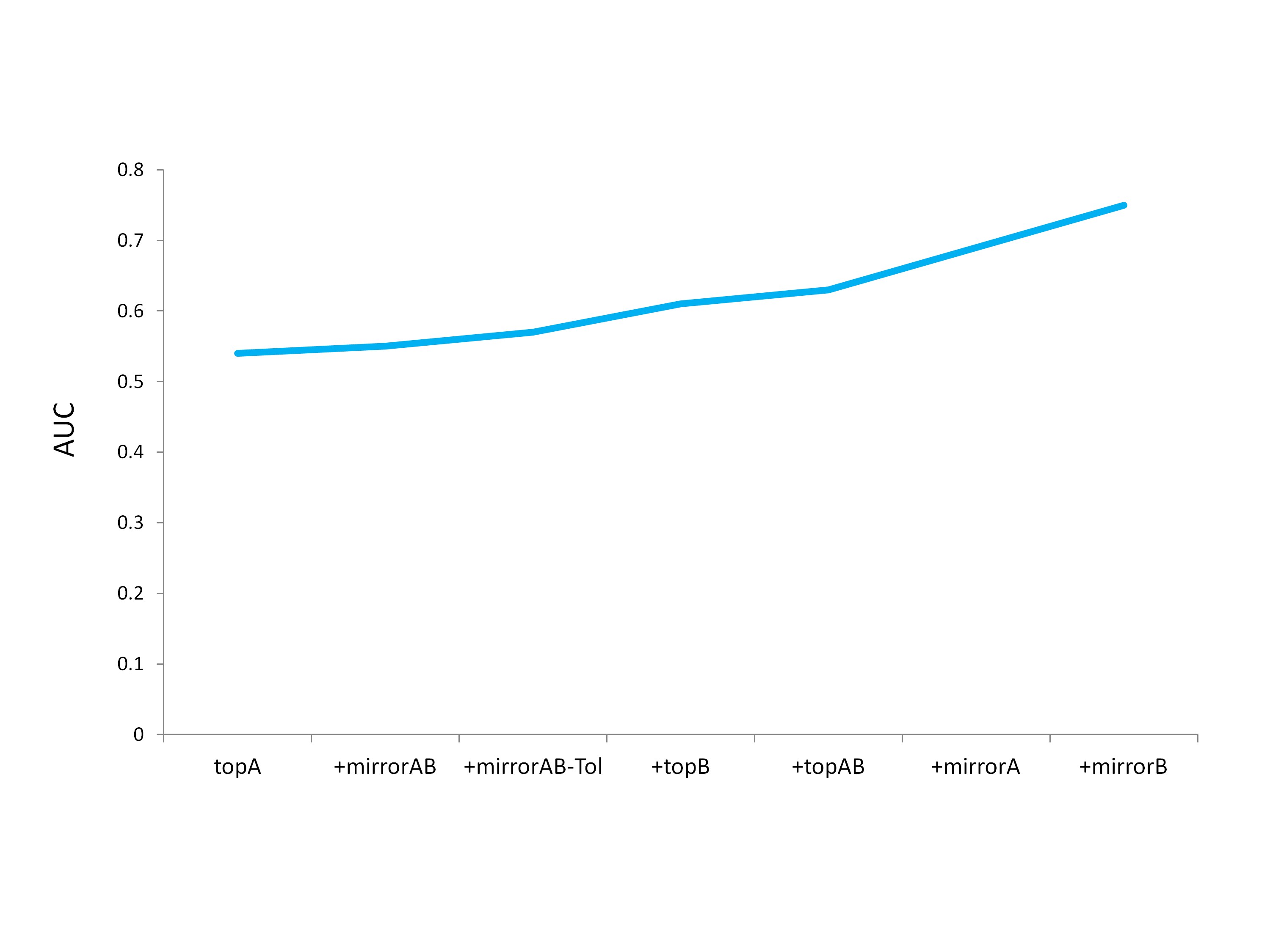

Supplement: S2 Fig — AUC values were calculated after running the RF classifier with different incremental combinations of the descriptors, starting from the most accurate and adding the next best one at a time. Here again the classifier yields the best performance when combining all the descriptors. (TIF) [file pcbi.1004067.s002.tif]
