# Supplementary material for: Exploring Bacterial Organelle Interactomes: A Model of the Protein-Protein Interaction Network in the Pdu Microcompartment
Source: PLoS Comput Biol. 2015 Feb 3;11(2):e1004067. doi: 10.1371/journal.pcbi.1004067 (PMC4315436; doi:10.1371/journal.pcbi.1004067)
Supplement: S1 Dataset — Protein-Protein interactions predicted by the RF classifier along with their respective mean probabilities (PPI in bold had P >0.7 and were used for the construction of the Pdu interaction network pictured in Fig. 3). (DOCX) [file pcbi.1004067.s003.docx]

**pduA|pduB 1 Parsons et al[81]**

**pduC|pduD 1 Bobik et al [22]**

**pduC|pduG 1**

**pduA|pduG 1**

**pduC|pduE 1 Bobik et al [22]**

**pduC|pduN 1**

**pduA|pduP 1 Fan et al [36]**

**pduE|pduF 0.9**

**pduG|pduH 0.9 Shibata et al[78]**

**pduA|pduC 0.9**

**pduJ|pduP 0.9 Fan et al [36]**

**pduA|pduJ 0.9 Parsons et al[81]**

**pduA|pduL 0.9**

**pduA|pduN 0.9 Parsons et al[81]**

**pduH|pduW 0.9**

**pduA|pduU 0.9 Parsons et al[81]**

**pduE|pduS 0.9**

**pduK|pduW 0.9**

**pduA|pduD 0.8**

**pduM|pduP 0.8**

**pduD|pduH 0.8**

**pduA|pduE 0.8**

**pduD|pduF 0.8**

**pduK|pduM 0.8 Parsons et al[81]**

**pduD|pduE 0.8 Bobik et al [22]**

**pduC|pduL 0.8**

**pduC|pduJ 0.8**

**pduD|pduO 0.8**

**pduC|pduP 0.8**

**pduG|pduL 0.8**

**pduG|pduJ 0.8**

**pduP|pduW 0.8**

**pduO|pduW 0.8**

**pduO|pduS 0.8 Cheng et al[100]**

**pduM|pduS 0.8**

**pduU|pduV 0.7**

**pduS|pduT 0.7 Parsons et al[99]**

**pduE|pduH 0.7**

**pduS|pduW 0.7**

**pduL|pduP 0.7**

**pduL|pduO 0.7**

**pduG|pduO 0.7**

**pduG|pduP 0.7**

**pduD|pduJ 0.7**

**pduA|pduK 0.7 Parsons et al[81]**

**pduG|pduK 0.7**

**pduD|pduT 0.7**

**pduG|pduU 0.7**

**pduC|pduW 0.7**

**pduJ|pduS 0.7**

**pduP|pduQ 0.7 Cheng et al [89]**

pduU|pduW 0.6

pduF|pduG 0.6

pduB|pduF 0.6

pduT|pduW 0.6

pduC|pduH 0.6

pduC|pduF 0.6

pduE|pduM 0.6

pduD|pduL 0.6

pduD|pduK 0.6

pduH|pduO 0.6

pduE|pduO 0.6

pduG|pduN 0.6

pduE|pduJ 0.6

pduF|pduL 0.6

pduC|pduS 0.6

pduF|pduW 0.6

pduD|pduW 0.6

pduC|pduV 0.6

pduB|pduW 0.6

pduC|pduU 0.6

pduG|pduQ 0.6

pduK|pduT 0.6

pduL|pduQ 0.6

pduM|pduW 0.6

pduN|pduQ 0.6

pduJ|pduW 0.6

pduL|pduU 0.6

pduW|pduX 0.5

pduN|pduP 0.5

pduN|pduO 0.5

pduD|pduG 0.5

pduA|pduF 0.5

pduJ|pduO 0.5

pduE|pduN 0.5

pduH|pduM 0.5

pduD|pduN 0.5

pduA|pduO 0.5

pduH|pduJ 0.5

pduE|pduL 0.5

pduF|pduK 0.5

pduE|pduV 0.5

pduE|pduU 0.5

pduD|pduQ 0.5

pduE|pduW 0.5

pduA|pduS 0.5

pduF|pduX 0.5

pduE|pduQ 0.5

pduH|pduT 0.5

pduE|pduT 0.5

pduF|pduS 0.5

pduA|pduW 0.5

pduN|pduU 0.5

pduO|pduV 0.5

pduN|pduW 0.5

pduM|pduX 0.5

pduO|pduT 0.5

pduL|pduW 0.5

pduP|pduT 0.5
