# Supplementary material for: Exploring Bacterial Organelle Interactomes: A Model of the Protein-Protein Interaction Network in the Pdu Microcompartment
Source: PLoS Comput Biol. 2015 Feb 3;11(2):e1004067. doi: 10.1371/journal.pcbi.1004067 (PMC4315436; doi:10.1371/journal.pcbi.1004067)
Supplement: S2 Dataset — (DOCX) [file pcbi.1004067.s004.docx]

STM2037

STM2038

STM2039

STM2040

STM2041

STM2042

STM2043

STM2044

STM2045

STM2046

STM2047

STM2048

STM2049

STM2050

STM2051

STM2052

STM2053

STM2054

STM2055

STM2056

STM2057

STM2058

STMUK_2067

STMUK_2068

STMUK_2069

STMUK_2070

STMUK_2071

STMUK_2072

STMUK_2073

STMUK_2074

STMUK_2075

STMUK_2076

STMUK_2077

STMUK_2078

STMUK_2079

STMUK_2080

STMUK_2081

STMUK_2082

STMUK_2083

STMUK_2084

STMUK_2085

STMUK_2086

STMUK_2087

STMUK_2088

SNSL254_A2214

SNSL254_A2215

SNSL254_A2216

SNSL254_A2217

SNSL254_A2218

SNSL254_A2219

SNSL254_A2220

SNSL254_A2230

SNSL254_A2231

SNSL254_A2232

SNSL254_A2233

SNSL254_A2234

SNSL254_A2235

SNSL254_A2221

SNSL254_A2222

SNSL254_A2223

SNSL254_A2224

SNSL254_A2225

SNSL254_A2226

SNSL254_A2227

SNSL254_A2228

SNSL254_A2229

CFSAN001921_06600

CFSAN001921_06595

CFSAN001921_06590

CFSAN001921_06585

CFSAN001921_06580

CFSAN001921_06575

CFSAN001921_06570

CFSAN001921_06565

CFSAN001921_06560

CFSAN001921_06555

CFSAN001921_06550

CFSAN001921_06545

CFSAN001921_06540

CFSAN001921_06535

CFSAN001921_06530

CFSAN001921_06525

CFSAN001921_06520

CFSAN001921_06515

CFSAN001921_06510

CFSAN001921_06505

CFSAN001921_06500

CFSAN001921_06495

CKO_00788

CKO_00789

CKO_00790

CKO_00791

CKO_00792

CKO_00793

CKO_00794

CKO_00795

CKO_00796

CKO_00797

CKO_00798

CKO_00799

CKO_00778

CKO_00779

CKO_00780

CKO_00781

CKO_00782

CKO_00783

CKO_00784

CKO_00785

CKO_00786

CKO_00787

ROD_21221

ROD_21241

ROD_21251

ROD_21261

ROD_21271

ROD_21281

ROD_21291

ROD_21301

ROD_21311

ROD_21321

ROD_21331

ROD_21341

ROD_21351

ROD_21361

ROD_21371

ROD_21381

ROD_21391

ROD_21401

ROD_21411

ROD_21421

ROD_21431

ROD_21441

Entcl_1760

Entcl_1759

Entcl_1758

Entcl_1757

Entcl_1756

Entcl_1755

Entcl_1754

Entcl_1753

Entcl_1752

Entcl_1751

Entcl_1750

Entcl_1749

Entcl_1748

Entcl_1747

Entcl_1746

Entcl_1745

Entcl_1744

Entcl_1743

Entcl_1742

Entcl_1741

Entcl_1740

Entcl_1739

ECUMN_2333

ECUMN_2334

ECUMN_2335

ECUMN_2336

ECUMN_2337

ECUMN_2338

ECUMN_2339

ECUMN_2340

ECUMN_2342

ECUMN_2343

ECUMN_2344

ECUMN_2345

ECUMN_2346

ECUMN_2347

ECUMN_2348

ECUMN_2349

ECUMN_2350

ECUMN_2351

ECUMN_2352

NRG857_10145

NRG857_10150

NRG857_10155

NRG857_10160

NRG857_10165

NRG857_10170

NRG857_10175

NRG857_10180

NRG857_10185

NRG857_10190

NRG857_10195

NRG857_10200

NRG857_10205

NRG857_10210

NRG857_10215

NRG857_10220

NRG857_10225

NRG857_10230

NRG857_10235

NRG857_10240

EFER_2007

EFER_2008

EFER_2009

EFER_2010

EFER_2011

EFER_2012

EFER_2013

EFER_2014

EFER_2016

EFER_2017

EFER_2018

EFER_2019

EFER_2020

EFER_2021

EFER_2022

EFER_2023

EFER_2024

EFER_2025

EFER_2026

EFER_2027

A79E_0908

A79E_0906

A79E_0905

A79E_0904

A79E_0903

A79E_0902

A79E_0901

A79E_0900

A79E_0899

A79E_0898

A79E_0897

A79E_0896

A79E_0895

A79E_0894

A79E_0893

A79E_0892

A79E_0891

A79E_0890

A79E_0889

A79E_0888

A79E_0887

KPK_0914

KPK_0913

KPK_0912

KPK_0911

KPK_0910

KPK_0909

KPK_0908

KPK_0907

KPK_0906

KPK_0905

KPK_0904

KPK_0903

KPK_0902

KPK_0901

KPK_0900

KPK_0899

KPK_0898

KPK_0897

KPK_0896

KPK_0895

KPK_0894

KPK_0893

Kvar_0868

Kvar_0867

Kvar_0866

Kvar_0865

Kvar_0864

Kvar_0863

Kvar_0862

Kvar_0861

Kvar_0860

Kvar_0859

Kvar_0858

Kvar_0857

Kvar_0856

Kvar_0855

Kvar_0854

Kvar_0853

Kvar_0852

Kvar_0851

Kvar_0850

Kvar_0849

Kvar_0848

Kvar_0847

Kvar_0846

Tola_1683

Tola_1684

Tola_1685

Tola_1686

Tola_1687

Tola_1688

Tola_1689

Tola_1690

Tola_1691

Tola_1692

Tola_1693

Tola_1694

Tola_1695

Tola_1696

Tola_1697

Tola_1698

Tola_1699

Tola_1700

Tola_1701

Tola_1702

Tola_1703

Tola_1704

Tola_1705

Tola_1706

Awo_c25910

Awo_c25900

Awo_c25890

Awo_c25880

Awo_c25870

Awo_c25860

Awo_c25850

Awo_c25840

Awo_c25830

Awo_c25820

Awo_c25810

Awo_c25800

Awo_c25790

Awo_c25780

Awo_c25770

Awo_c25760

Awo_c25750

Awo_c25740

ELI_4085

ELI_4084

ELI_4083

ELI_4082

ELI_4081

ELI_4080

ELI_4079

ELI_4078

ELI_4077

ELI_4076

ELI_4075

ELI_4074

ELI_4073

ELI_4072

ELI_4071

ELI_4070

lse_1020

lse_1021

lse_1022

lse_1023

lse_1024

lse_1025

lse_1028

lse_1029

lse_1026

lse_1027

lse_1030

lse_1031

lse_1032

lse_1033

lse_1034

lse_1035

lse_1036

lse_1037

lse_1038

lse_1039

lse_1040

lse_1041

lse_1042

lse_1043

lse_1044

lse_1045

lse_1046

lse_1047

lse_1048

LIV_1074

LIV_1075

LIV_1076

LIV_1077

LIV_1078

LIV_1079

LIV_1080

LIV_1081

LIV_1083

LIV_1084

LIV_1085

LIV_1086

LIV_1087

LIV_1088

LIV_1089

LIV_1090

LIV_1091

LIV_1092

LIV_1093

LIV_1094

LIV_1095

LIV_1096

LIV_1097

LIV_1098

LIV_1099

LIV_1100

LIV_1101

LIV_1102

LMRG_00585

LMRG_00586

LMRG_00587

LMRG_00588

LMRG_00589

LMRG_00590

LMRG_00591

LMRG_00592

LMRG_00593

LMRG_00594

LMRG_00595

LMRG_00596

LMRG_00597

LMRG_00598

LMRG_00599

LMRG_00600

LMRG_00601

LMRG_00602

LMRG_00603

LMRG_00604

LMRG_00605

LMRG_00606

LMRG_00607

LMRG_00608

LMRG_00609

LMRG_00610

LMRG_00611

LMRG_00612

LMRG_00613

TherJR_0613

TherJR_0614

TherJR_0615

TherJR_0616

TherJR_0617

TherJR_0618

TherJR_0619

TherJR_0620

TherJR_0623

TherJR_0624

TherJR_0625

TherJR_0626

TherJR_0627

TherJR_0628

TherJR_0629

TherJR_0630

TherJR_0631

TherJR_0632

TherJR_0621

TherJR_0622

TherJR_0633

HMPREF9154_2974

HMPREF9154_2975

HMPREF9154_2976

HMPREF9154_2977

HMPREF9154_2978

HMPREF9154_2979

HMPREF9154_2980

HMPREF9154_2981

HMPREF9154_2982

HMPREF9154_2983

HMPREF9154_2984

HMPREF9154_2985

HMPREF9154_2986

HMPREF9154_2987

HMPREF9154_2988

HMPREF9154_2989

HMPREF9154_2990

HMPREF9154_2991

Anamo_0110

Anamo_0111

Anamo_0112

Anamo_0113

Anamo_0114

Anamo_0115

Anamo_0116

Anamo_0117

Anamo_0118

Anamo_0119

Anamo_0120

Anamo_0121

Anamo_0122

Anamo_0123

Anamo_0124

Anamo_0125

Anamo_0126

Anamo_0127

Anamo_0128

Anamo_0129

Anamo_0130

Geoth_1925

Geoth_1926

Geoth_1927

Geoth_1928

Geoth_1929

Geoth_1930

Geoth_1931

Geoth_1932

Geoth_1933

Geoth_1934

Geoth_1935

Geoth_1936

Geoth_1937

Geoth_1938

Geoth_1939

Geoth_1941

YE2727

YE2728

YE2729

YE2730

YE2731

YE2732

YE2733

YE2734

YE2735

YE2736

YE2737

YE2738

YE2739

YE2740

YE2741

YE2742

YE2743

YE2744

YE2745

YE2746

YE2747

YE2748

YE2749

YE2750

LMOATCC19117_1141

LMOATCC19117_1142

LMOATCC19117_1143

LMOATCC19117_1144

LMOATCC19117_1145

LMOATCC19117_1146

LMOATCC19117_1147

LMOATCC19117_1148

LMOATCC19117_1149

LMOATCC19117_1150

LMOATCC19117_1151

LMOATCC19117_1152

LMOATCC19117_1153

LMOATCC19117_1154

LMOATCC19117_1155

LMOATCC19117_1156

LMOATCC19117_1157

LMOATCC19117_1158

LMOATCC19117_1159

LMOATCC19117_1160

LMOATCC19117_1161

LMOATCC19117_1162

LMOATCC19117_1163

LMOATCC19117_1164

LMOATCC19117_1165

LMOATCC19117_1166

LMOATCC19117_1167

LMOATCC19117_1168

LMOATCC19117_1169

LMOATCC19117_1170

Ilyop_2342

Ilyop_2343

Ilyop_2344

Ilyop_2345

Ilyop_2346

Ilyop_2347

Ilyop_2348

Ilyop_2349

Ilyop_2350

Ilyop_2351

Ilyop_2352

Ilyop_2353

Ilyop_2354

Ilyop_2355

Ilyop_2356

Ilyop_2357

Ilyop_2358

Ilyop_2359

Ilyop_2360

Ilyop_2361

PFREUD_08970

PFREUD_08980

PFREUD_08990

PFREUD_09000

PFREUD_09010

PFREUD_09020

PFREUD_09030

PFREUD_09040

PFREUD_09050

PFREUD_09060

PFREUD_09070

PFREUD_09080

PFREUD_09090

PFREUD_09100

PFREUD_09110

PFREUD_09120

PFREUD_09130

LAR_1640

LAR_1639

LAR_1638

LAR_1637

LAR_1636

LAR_1635

LAR_1634

LAR_1633

LAR_1632

LAR_1631

LAR_1630

LAR_1629

LAR_1628

LAR_1627

LAR_1626

LAR_1625

LAR_1624

LAR_1623

LAR_1622

LAR_1621

LAR_1620

LAR_1619

LAR_1618

LAR_1617

LAR_1616

SSON_2057

SSON_2058

SSON_2059

SSON_2060

SSON_2061

SSON_2062

SSON_2063

SSON_2064

SSON_2065

SSON_2066

SSON_2067

SSON_2068

SSON_2069

SSON_2070

SSON_2071

SSON_2072

SSON_2073

SSON_2074

SSON_2075

SSON_2076

PECL_1367

PECL_1368

PECL_1369

PECL_1370

PECL_1371

PECL_1372

PECL_1373

PECL_1374

PECL_1375

PECL_1376

PECL_1377

PECL_1378

PECL_1379

PECL_1380

PECL_1381

PECL_1382

PECL_1383

PECL_1384

PECL_1385

lwe1100

lwe1101

lwe1102

lwe1103

lwe1104

lwe1105

lwe1108

lwe1109

lwe1106

lwe1107

lwe1110

lwe1111

lwe1112

lwe1113

lwe1114

lwe1115

lwe1116

lwe1117

lwe1118

lwe1119

lwe1120

lwe1121

lwe1122

lwe1123

lwe1124

lwe1125

lwe1126

SPUCDC_0862

SPUCDC_0861

SPUCDC_0860

SPUCDC_0859

SPUCDC_0858

SPUCDC_0857

SPUCDC_0856

SPUCDC_0855

SPUCDC_0854

SPUCDC_0853

SPUCDC_0852

SPUCDC_0851

SPUCDC_0849

SPUCDC_0848

SPUCDC_0847

SPUCDC_0846

SPUCDC_0845

SPUCDC_0844

SPUCDC_0843

SPUCDC_0842

HMPREF0409_01346

HMPREF0409_01347

HMPREF0409_01348

HMPREF0409_01349

HMPREF0409_01350

HMPREF0409_01351

HMPREF0409_01352

HMPREF0409_01353

HMPREF0409_01354

HMPREF0409_01355

HMPREF0409_01356

HMPREF0409_01357

HMPREF0409_01358

HMPREF0409_01359

HMPREF0409_01360

HMPREF0409_01361

GY4MC1_1859

GY4MC1_1860

GY4MC1_1861

GY4MC1_1862

GY4MC1_1863

GY4MC1_1864

GY4MC1_1865

GY4MC1_1866

GY4MC1_1867

GY4MC1_1868

GY4MC1_1869

GY4MC1_1870

GY4MC1_1871

GY4MC1_1872

GY4MC1_1873

GY4MC1_1875

GY4MC1_1876

Halsa_0982

Halsa_0983

Halsa_0984

Halsa_0985

Halsa_0986

Halsa_0987

Halsa_0988

Halsa_0989

Halsa_0990

Halsa_0991

Halsa_0992

Halsa_0993

Halsa_0994

Halsa_0995

Halsa_0996

Halsa_0997

Halsa_0998

Halsa_0999

Halsa_1000

Halsa_1001

Halsa_1002

Halsa_1003

Halsa_1004
